# Selections

**Published:** 1871-04

**Authors:** 


### Selections.

*On Mechanical Means of Relief in Certain Cases of Inflammatory Abdominal Effusions.* By JAMES RISDON BENNETT, M.D.

In several recent numbers of the *Practitioner* reference has been made to the successful treatment of tympanitic distension of the belly by puncturing the intestines through the abdominal walls. Should further experience prove that this may be as safely done on the human body as we know it is in the case of animals, there can be no doubt that, in various forms of abdominal disease, great advantage may be derived from such a mode of relief to the distress and danger attending extreme distension of the belly from tympanitis. The intelligent practitioner will be able to judge how far puncturing the intestine may be admissible in any given case, just as he now does of the dangers attending the ordinary operation of paracentesis abdominis. But we must wait for further experience before any definite rules can be laid down regarding the circumstances in which we should be justified in having recourse to an operation manifestly not devoid of serious risk. In the meantime, every instance in which the operation has been resorted to deserves to be recorded. For this reason, therefore, I have thought that the interesting case I am about to detail might be a not unacceptable contribution to the *Practitioner*. It is certainly a remarkable example of the bold and successful employment of mechanical means of relief in one of the most common forms of abdominal disease falling under the care of the physician, and will, I am sure, be read with interest by every practical physician.

The war having broken out just as I was preparing to take my continental holiday, and so interfering with my projected route, I determined to visit Russia by the Baltic and return by Stockholm and Christiania. I thus had an opportunity, this year, of seeing something of the Northern hospitals, although, as a rule, I think it desirable to leave physic behind me and eschew hospitals and matters medical when on my holiday. There are, however, both in Russia and Scandinavia, things worthy of notice by British practitioners.

At Christiania I was introduced to Dr. Stabell, a most accomplished and able physician attached to the principal hospital of that place. Among other cases of unusual interest (including both the forms of Norwegian leprosy, the tubercular and the anæsthetic), he showed me the young man whose case I am about to describe, from the full and careful notes kindly furnished me by Dr. Stabell.

A medical student, aged 24, entered the Christiania Hospital, as a patient, on the 17th March, 1870. Twelve days previously he first felt unwell, having headache and loss of appetite. He was not, however, obliged to keep his bed, but even went out occasionally. Whilst out walking on the 13th March he was seized with a sudden pain in the hypogastrium, which became tender on pressure, and he thought somewhat swollen. He went to the theatre the same evening, but was obliged to leave in consequence of the pain becoming worse. The following day he went out again, and accidentally got a kick on the belly by which the pain was so much aggravated that he was obliged to take a carriage home and go to bed.

17*th.*—On admission to the ward of the hospital his condition was as follows: Loss of appetite, thirst, some sickness and vomiting, the latter having existed two days, together with diarrhœa. Yesterday he had seven fluid evacuations. Pain in the hypogastric region, not severe when quiet, but increased by motion or coughing. The whole abdomen tense and swollen, not very tender on pressure, except between the umbilicus and the left iliac crest, where there is extreme tenderness. Percussion dull from the umbilicus to the symphysis. Fluctuation not very distinct. Sound on percussion tympanitic from the sixth rib to the navel. Heart and liver normal in situation. Lungs apparently normal, but he has some cough and dyspnœa. He lost a brother from phthisis last year. Pulse 96. Temperature 37·8 centigrade. He was ordered tinct. thebaicæ and acid. hydrocyan. dil., āā m v. every two hours; a wet bandage to the abdomen, and hypodermic injection of morphia in the evening.

For some days his condition was much the same. On the 25th the dullness on percussion extended to five fingers' breadth above the navel. There were daily two or three loose motions, and the occasional passing of flatus gave him considerable relief. The pulse, however, fell to between 70 and 80. The temperature ranged between 37 and 38 centigrade, and the tongue was dry.

31*st.*—Eight leeches were applied to the abdomen, and an enema administered. The pain was for a short time relieved by the leeches, and still greater relief followed a copious action of the bowels from the enema. The pain and some tenesmus, however, soon returned, and he had sickness and bilious vomiting. Two morphia injections afforded very little relief. The upper part of the belly became greatly distended from tympanitis, and there was more prostration. He was ordered opii gr. $\frac{1}{2}$ and calomel gr. 1 every two hours. An œsophageal tube was introduced into the rectum, through which some air and fluid motion passed.

*April 3d.*—The distension of the transverse colon had become very great, and there was more dyspnœa. An exploring trochar was therefore introduced into the most prominent portion of the transverse colon, and a good deal of air evacuated with considerable relief. The following day, however, the distension was as great as before, and it was now determined to evacuate the fluid by tapping with an ordinary trochar. The abdomen was punctured midway between the umbilicus and the symphysis pubis, and four "potter" of yellowish semipuriform fluid removed (the Norwegian measure "pot" contains 1,000 c. c.). It was not attempted to remove the whole of the fluid, the trochar being withdrawn so soon as the fluid began to flow slowly. The size of the abdomen was considerably reduced, but the distension of the transverse colon remained the same. The following day the patient felt better, but complained of pain in the left leg, which had become œdematous. There was tenderness along the course of the large vessels and some tumefaction of the veins. Large water enemata were administered daily, which removed fæculent matter and a good deal of flatus. Some pain and soreness existing around the puncture, an ice-bag was applied. The following day he vomited occasionally, but the swelling of the leg somewhat subsided.

*5th.*—Temperature 38—37·1. Pulse 88. He continues the use of lukewarm water enemata.

*8th.*—Some return of appetite and more sleep. Tongue less dry; two to four semi-fluid motions a day.

*15th.*—Effusion extends to two fingers' breadth above the navel, and paracentesis was again performed, by which over two "potter" of thick pus were evacuated.

*16th.*—The percussion dulness extends from the symphysis to two fingers' breadth below the navel. ℞ Extracti cinchonæ, tinct. nucis vomicæ, āā ʒ j, aquæ fœniculi ℥ j, M: a teaspoonful to be taken three times a day. The effusion speedily accumulated again, and on the 25th he was tapped a third time, and 3¾ "potter" of somewhat offensive pus were withdrawn.

*26th.*—Pulse 72.

*28th.*—Pulse 72, temperature 36·6. Bowels act two to three times daily. Motions more solid.

*May* 4th.—Some œdema of the scrotum; this, however, on the 7th had nearly disappeared, but the feet and ankles were a little œdematous; percussion dull to the navel, but less towards the iliac fossæ, and towards the centre the abdomen is more prominent and resistent where the sense of fluctuation is distinct. On the 8th all medicine was omitted.

*12th.*—Abdomen again tapped, and 4½ "potter" of offensive pus evacuated. A day or two after this he was ordered a tablespoonful of chalybeate cinchona wine three times a day.

*16th.*—Some strangury and a little pus in the urine.

*20th.*—Effusion again increasing up to the navel, with distinct fluctuation in the iliac fossæ and a little hard infiltration in the abdominal wall, in the vicinity of the puncture. He, however, feels better; was up in a chair for two hours yesterday, and had one copious natural action of the bowels.

*21st.*—Tapped with a larger-sized trochar, and a "pot" and a half of pus evacuated. Through the canula of this large trochar, another double canula was introduced, and by means of an india-rubber tube and an elevated reservoir of water, the abdominal cavity was washed out with a weak solution of common salt in warm water, till the water from the abdomen ran away quite clear.

*22d.*—Passed a comfortable night after a morphia hypodermic injection. Bowels acted once naturally. The percussion note is now clear almost to the symphysis pubis. Urine clearer, and less frequent desire to pass water.

*24th.*—Has been up again.

*27th.*—Urine sedimentous, feebly alkaline, presenting, under the microscope, some pus-cells and vibriones. Some discharge, now and then, from the puncture, which remains open. A drainage tube was introduced into the abdomen, to the extent of five inches, and left in the orifice; and this he continued to wear.

*June 23d.*—Was out in the garden; still wears the drainage-tube in the abdomen. Urine continues alkaline, with a muco-purulent sediment. Appetite, aspect, and strength much improved.

*July 17th.*—Continued in much the same state, the discharge from the fistula varying in amount and character, the pus being sometimes offensive. The urine has maintained the same character. He has felt well and been out in the garden daily. Yesterday, however, he had a rigor followed by perspiration and headache, the temperature rising to 39·4. This morning he felt better, but at ten o'clock there was a recurrence of rigor with a temperature of 41. Pulse 134. The drainage-tube cannot be introduced beyond half an inch; but, after an exploration with a probe, the tube again passed in to the extent of three inches, when some offensive matter was discharged. Ten grains of quinine were given in the morning.

On the 19th July the temperature had again fallen to 36, and the pulse to 56. With the exception of the cystitis, which did not trouble him much, as there was no pain and scarcely any tenesmus, and for which he took first decoct. uvæ ursi and subsequently decoct. pareiræ, he was now pretty well again. On the 27th July, however, he had another severe rigor early in the morning, the temperature rising to 41° and the pulse to 140. He now left off all other medicine and began taking quinine. There being some tenderness around the fistulous opening, a wet bandage was applied.

*July 28th.*—He was again pretty well, the temperature having fallen to 36·6 and the pulse to 60, and the tenderness of the orifice had vanished.

Thus far I have given the history as kindly furnished me by Dr. Stabell. When I saw the patient in August he was sitting up in his room, looking pale and pulled down, as though he had had a severe illness, but he was cheerful and free from fever or any discomfort. His pulse was quiet and of fair strength, the tongue clean, the appetite good, and the bowels regular. The abdomen was soft and pliant, of natural form and size, and devoid of all tenderness on manipulation. He was still wearing the drainage tube, which he withdrew and replaced to show me the ease with which he could do so, and the depth of three to four inches to which it passed within the abdominal cavity. I could not detect any local hardness or swelling, and the abdomen had its natural, soft, elastic feel.

He left the hospital to go home on the 23d August, feeling in all respects well, but still wearing the tube, as there was still sufficient discharge to make it imprudent to allow the opening to close.

Before drawing any conclusions from this very interesting and instructive case for future therapeutic guidance, it is important to satisfy ourselves as to the real nature of the case. That there was a low form of peritoneal inflammation cannot admit of doubt; but was it, in the first instance, general peritonitis or localized in the lower pelvic region? And, subsequently, was the lower abdominal region shut off from the upper by adhesions? Thus did the saline injection wash out the whole abdominal cavity, or only a limited sac; and did the drainage-tube pass into the abdominal cavity, or only into such a sac? That at one time a very considerable amount of fluid existed in the abdomen cannot be doubted; and this evidently accumulated, in the first instance, in the lower belly. The tympanitic distension of the colon and the deranged state of the intestinal functions would seem to imply that the inflammatory action was not at first very limited. The slight degree of febrile disturbance and low temperature, except on the temporary occasions mentioned, may, perhaps, in the opinion of some persons, rather militate against the notion of general peritonitis. But it is not, I think, at all uncommon to find an almost entire absence of fever in the low forms of peritonitis seen in tubercular subjects.

But whatever view may be taken of the case, the treatment so successfully adopted appears deserving of commemoration and of careful consideration in cases of inflammatory effusions into the abdomen, attaining to such a degree, and lasting so long, as to threaten life. The patient in this case expressed to me the great relief that he obtained by the evacuation of flatus from the colon, and believes that his life even was thus saved.

It is now pretty well established that many cases of empyema do best when a free permanent opening in the thoracic walls allows of continuous discharge of the purulent secretion. In some important respects the abdomen is more favorably circumstanced for such a procedure than the thorax. Nature herself adopts such a mode of relief, and I have seen cases of extensive tubercular peritonitis where life appeared to have been considerably prolonged by the drainage of the effusion through a spontaneous opening through the abdominal walls. I remember having had under my care, many years ago, a lad who was the subject eventually of universal peritoneal inflammation and adhesions. In the early stage of his illness he had symptoms of obstructed bowel and a tumor in the right groin distended under the impulse of coughing. For this he had been taken to one of our London hospitals, and was operated on for inguinal hernia From the opening thus made there was a continuous discharge for some time, with very considerable temporary improvement in his condition.

How far the washing out of the peritoneal cavity with the weak saline solution may have contributed to the favorable issue of the case detailed, it may be difficult to say. But it is a practice occasionally resorted to with advantage, I am told, after operations for ovariotomy.—*Anstie's Practitioner.*

---

*On the Treatment of Typhoid Fever by Cold Water Baths.* By J. H. Tyndale, M. D., House Surgeon to the German Hospital, N. Y.

Late researches have developed a method of cure, by the application of which we are enabled to reduce the rate of mortality among typhoid fever patients by from three to five per cent. This method is the treatment of typhoid fever by cold water baths, practiced in the last century, but lately revived by Brand, of Stettin, and since submitted to a scientific and practical test by a great number of physicians on the continent of Europe. The verdict in favor of this method of treatment of typhoid fever on rational principles has been universal, and attested by numerous and responsible clinical reports, comprising many thousands of cases.

The cold water treatment cannot prevent the natural course of typhoid fever. The natural phases, with their peculiar anatomical changes, will appear in an undiminished degree. Cases of death from perforation of the bowel or hæmorrhage have not been diminished any more than if no treatment at all had been prescribed. The cases of death from these causes, however, has always been counted as an incomparably small fragment of the total mortality among typhoid fever patients. The principal source of danger for the patient is the *fever heat*, either directly or indirectly, and we are enabled to reduce this unnatural elevation of the temperature of the body, and thereby the degree of danger to the patient, by cold baths. The dangerous effect of fever heat in typhoid fever, as manifested by the so-called nervous manifestations: delirium, sleeplessness in the firsts tage, listlessness in the further course of the disease, depends entirely upon the continued unnatural temperature of the body. Death from these causes has never revealed any anatomical lesions. *Such symptoms will not manifest themselves if by timely, energetic, and oft-repeated withdrawal of warmth, a cooling off of the body is effected.* The patient remains sensible, and involuntary voiding of fæces and urine is of rare occurrence.

Not merely the psychical manifestations, but also the other functions of the nervous system, as well as muscular activity, are more or less impaired by fever heat. The paralyzing influence is shown by the feebleness of respiration, giving rise to the fatal collapse and inflammatory conditions of the lungs. The fever heat checks or limits excretion, interferes in this way with the functions of digestion, produces loss of appetite, and stops the supply of natural material necessary to supply the rapid waste of tissue.

All these sequels of the feverish overheating of the body are observed to manifest themselves in a lesser degree by the use of cold water baths. Thus, all competent observers are agreed upon the fact that the patient never loses his appetite; on the contrary, takes food during the whole course of the fever, so that extreme emaciation will not ensue, and the patient regains his strength in a shorter space of time from the period of convalescence. Bed sores, so frequent and unavoidable in typhoid fever, have been but rarely observed during the cold water treatment. In short, all secondary complications of typhoid fever have been totally excluded by this method of treatment, and the whole course of the disease has been completed in four weeks.

The general rules to be observed in administering cold water baths are the following:

1. The necessary reduction of temperature is best and most rapidly effected by immersing the whole body.

2. The water should be as cold as can be had.

3. The patient should be bathed as often as the temperature of his body, measured in the rectum, rise to 40° C. (about 104° Fahr.) Since the intensity of the manifestations of disease vary much, it may occur that in one case one or two baths in the twenty-four hours will suffice, whereas, in another, as many as twelve or sixteen will be required in the same space of time.

4. The length of time for each bath must be governed on the one hand by the degree of fever heat, on the other by the temperature of the water used. On the whole it will be found that in a bath varying from 5° to 10° C., an immersion of from seven to ten minutes will suffice. Should the temperature of the water be above 10° C., the bath is to be continued for fifteen minutes, and if above 15° C., still longer.

No attention needs to be paid to the seeming discomfort of the patient, manifested by complaints, nor to the chill often occurring during the bath, and continuing for sometime afterward, as they are of no consequence.

5. After the bath, the patient should be carefully wiped dry (not rubbed), especially his feet and toes. If the water has been of very low temperature, the feet may be enveloped in warm cloths, as many patients complain of pain in the feet after a very cold bath.

Opinions differ as to whether it is best to immerse the patient in a cold bath (say of 10° C.) at once, or to have the water at a temperature more nearly the same as that of the body, and effect a gradual reduction by a slow addition of cold water. Niemeyer, who may be considered the best authority upon the subject, is in favor of a gradual reduction. With due deference to this opinion, however, I must say that repeated trials have satisfied me that by a sudden immersion in cold water two advantages are gained —1st, the reduction of temperature will be greater, more nearly approximating the normal temperature of the body; 2d, less time will be required, and consequently the patient will be less annoyed. In the cases under our observation we have found from one-half to two hours after sudden immersion the temperature reduced to 38.5° C. (normal), when before the bath it had been from 40° to 40.5° C.

When the temperature of the body has not been above 39.5° C., we have been in the habit of enveloping the patient in wet cold sheets for fifteen minutes. In other cases in which it was desirable to move the patient as little as possible, we have resorted to a sponge-bath of cold water and vinegar. Both methods produce a limited decrease of temperature, not exceeding one degree.

The antifebrile effect of cold water baths will be materially aided by large doses of quinine. From eight to ten grains of sulphate of quinine, administered every second evening, will surely obviate the necessity of frequent baths on the following day. Large doses, more frequently given, are apt to disagree, as also to lose their effect.

The *thermometer* is indispensable as an aid to the cold water treatment, as without it this method would lack the necessary safety in its application. The rectum is undoubtedly the best point of observation of the thermometer. In five minutes after the introduction of the bulb, the mercury will have reached its maximum height, and no disturbing influence can injure the correctness of observation, as is often the case in the introduction of the bulb into the axilla.

The severer the case, the oftener should thermometrical observations be made. In mild cases, in which even the evening temperature (always higher than the morning temperature) does not exceed 40° C., two or three observations may suffice; whereas, in severer ones, this should be done every two hours day and night, in order not to miss the right time for the repetition of the bath.—*St. Louis Medical and Surgical Journal.*

